# Murine metabolic HFpEF is associated with altered mitochondrial substrate handling and S-nitrosylation remodeling

**DOI:** 10.1016/j.redox.2026.104349

**Published:** 2026-08-12

**Authors:** Huihui Li, Hanyu Cui, Daiyu Wang, Florian Leuschner, Joerg Heineke, Jiong Hu, Sofia-Iris Bibli

**Affiliations:** a Department of Vascular Dysfunction, European Center for Angioscience (ECAS), Medical Faculty Mannheim, Heidelberg University, Mannheim, Germany; b Division of Cardiology, Department of Internal Medicine, Tongji Hospital, Tongji Medical College, Huazhong University of Science and Technology, Wuhan, China; c Department of Cardiology, The First Affiliated Hospital of Henan University, School of Medicine, Henan University, Kaifeng, China; d Department of Histology and Embryology, School of Basic Medicine, Tongji Medical College, Huazhong University of Science and Technology, Wuhan, China; e Department of Cardiology, Internal Medicine III, Heidelberg University Hospital, Heidelberg, Germany; f German Center of Cardiovascular Research (DZHK), Partner site Heidelberg-Mannheim, Germany; g Department of Cardiovascular Physiology, European Center for Angioscience (ECAS), Medical Faculty Mannheim, Heidelberg University, Mannheim, Germany; h Helmholtz-Institute for Translational AngioCardioScience (HI-TAC) of the Max Delbrück Center for Molecular Medicine in the Helmholtz Association (MDC) at Heidelberg University, Heidelberg, 69117, Germany; i Sino-German Laboratory of CardioPulmonary Science, Tongji Medical College, Huazhong University of Science and Technology, Wuhan, China; j Division of Cardiovascular Surgery, Tongji Hospital, Tongji Medical College, Huazhong University of Science and Technology, Wuhan, China

## Abstract

Heart failure with preserved ejection fraction (HFpEF) is a heterogeneous condition with incompletely defined myocardial mechanisms. Here, using a two-hit murine model of cardiometabolic HFpEF induced by high-fat diet and endothelial nitric oxide synthase inhibition, we define a mitochondrial metabolic phenotype characterized by altered substrate handling, redox stress, and S-nitrosylation remodeling. While global proteomic changes were modest, metabolomic profiling revealed selective remodeling of tricarboxylic acid cycle intermediates, increased dicarboxylic acids, and altered redox-associated metabolites, consistent with mitochondrial metabolic and redox imbalance in this experimental setting. S-nitrosylation proteomics demonstrated a highly organized and bidirectional remodeling pattern affecting proteins involved in fatty acid/lipid metabolism, carbohydrate metabolism, mitochondrial energy metabolism, amino acid and organic acid metabolism, nucleotide/co-factor metabolism, and redox defense. Stable isotope tracing showed reduced glucose-derived and increased palmitate-derived acetyl-CoA in HFpEF, whereas Na-βHB reduced palmitate contribution and increased βHB-derived acetyl-CoA without restoring glucose contribution, indicating substrate redistribution and preserved ketone oxidation. Na-βHB supplementation increased oligomycin-sensitive respiration in freshly prepared left ventricular tissue, partially normalized selected TCA-cycle intermediates, reduced mitochondrial ROS and the NADH/NAD^+^ ratio, restored the GSH/GSSG ratio, and improved diastolic function without altering ejection fraction. Together, these findings define a redox-sensitive mitochondrial metabolic state in the HFD/l-NAME model and identify ketone supplementation as a partial metabolic rescue strategy in this context. At the same time, these findings highlight an important limitation of the murine HFD/l-NAME model, which should be interpreted as an experimental system for studying high-fat-induced cardiometabolic stress rather than as a metabolic equivalent of human HFpEF.

## 1 Introduction

Heart failure with preserved ejection fraction (HFpEF) remains mechanistically elusive and therapeutically challenging [1,2]. Although metabolic stress is a major contributor to HFpEF in a substantial proportion of patients, the mechanisms linking obesity, insulin resistance, and nitric oxide dysregulation to impaired cardiac function remain unclear [2]. To investigate these mechanisms, several experimental models have been developed [3], among which the high-fat diet/l-NAME two-hit model is widely used because it recapitulates not only preserved ejection fraction, diastolic dysfunction and reduced exercise capacity, but also the systemic metabolic and inflammatory milieu, including obesity, glucose intolerance, hypertension, and nitric oxide dysregulation, that characterizes the clinical cardiometabolic HFpEF endotype [4].

Mitochondrial dysfunction has emerged as a recurring feature in HFpEF, but the basis of this dysfunction requires further investigation [5]. Emerging evidence suggests that regulatory events at the level of protein function may contribute to the pathophysiology of HFpEF [6,7]. In this disease context, cardiac mitochondrial proteins are susceptible to acute post-translational modifications (PTMs) and redox-dependent regulatory changes. These processes can interact bidirectionally with reactive oxygen species (ROS), thereby disrupting bioenergetic efficiency and amplifying mitochondrial stress [8]. S-nitrosylation is of particular interest in this context because it can directly modulate enzymatic activity, substrate utilization, and redox buffering [9]. We therefore combined global proteomics, S-nitrosylation proteomics, and metabolomics in a two-hit murine model of cardiometabolic HFpEF. Using this approach, we define a model-specific phenotype characterized by limited proteomic remodeling but pronounced metabolic and S-nitrosylation changes affecting mitochondrial and metabolic pathways. We further show that altering mitochondrial substrate utilization partially improves mitochondrial and functional readouts in this context. Together, these findings support the use of the HFD/l-NAME system as a model of high-fat-induced cardiometabolic stress responses, while recognizing that its metabolic organization may differ from that of human HFpEF.

## 2 Methods and data availability

### 2.1 Data availability

The mass spectrometry proteomics data have been deposited to the ProteomeXchange Consortium via the PRIDE partner repository with the dataset identifier PXD075362.

### 2.2 Animal studies

Metabolic HFpEF was induced and functionally characterized as described previously [4]. Briefly, male C57BL/6 N mice aged 8–12 weeks were used to maintain consistency with the originally established HFD/l-NAME HFpEF model. Because the present study was discovery-oriented and not designed or powered to assess sex-specific differences, only male mice were included. Mice were subjected to a two-hit protocol consisting of a high-fat diet containing 60% calories from fat and l-NAME administered in the drinking water at 0.5 g/L for 12 weeks. At the end of experiments, mice were anesthetized using 4% isoflurane in air and subsequently euthanized by decapitation. Then the hearts were rapidly excised. The great vessels and atrial appendages were removed, and the ventricular tissue was immediately snap-frozen in liquid nitrogen. Afterwards, frozen ventricular tissue was thoroughly pulverized and mixed to generate a homogeneous tissue powder. The resulting powder was then divided by weight into three aliquots for global proteomics, metabolomics and S-nitrosylation proteomics. For the treatment experiment, sodium DL-β-hydroxybutyrate (Na-βHB; Cat. No. 54965, Sigma-Aldrich) was added in the drinking water since the first day of diet initiation: 2% w/v ≈ 80–120 mg/day intake. Transthoracic echocardiography was performed with ultrasonography using a Vevo3100 system (Visualsonics, Toronto, Canada) as previously described [10]. Briefly, echocardiography was performed under light isoflurane anesthesia maintained at 1.0–1.5% and adjusted to maintain a heart rate of approximately 420–440 beats/min. Mice were not mechanically ventilated and temperature was monitored with a rectal thermometer. Cardiovascular parameters were analyzed using the Vevo LAB desktop software. Samples were collected following 16 hours of fasting for subsequent molecular analysis. Exercise capacity was assessed using a treadmill exhaustion test as previously described [11]. In brief, mice were acclimated to a rodent treadmill for three days prior to testing. A progressive maximal exercise test was performed in which treadmill speed was gradually increased until a maximum of 18 meters/minute was reached. Mice ran until exhaustion, defined as remaining on the shock grid for >5 second without resuming running. Total running time and distance were recorded. All animals were housed in conditions that conform to the Guide for the Care and Use of Laboratory Animals published by the National Institutes of Health (publication no. 85-23). The University Animal Care Committee and the Federal Authority for Animal Research at Tongji Medical College approved the study. Age-, sex-, and strain-matched animals (littermates) were used throughout the studies.

### 2.3 Global proteomics and S-nitrosylation proteomics

For global proteomics, tryptic peptides were desalted and analyzed by data-independent acquisition (DIA) LC–MS/MS on an Orbitrap Astral mass spectrometer coupled to a Vanquish Neo UHPLC system (Thermo Fisher Scientific) by Shanghai Bioprofile Technology Co., Ltd. Raw data were searched and quantified using Spectronaut, and the resulting protein- and modification-level quantitative matrices were median-normalized before downstream statistical analysis.

For S-nitrosylation proteomics, heart tissues were lysed in SDS-containing buffer, and free thiols were blocked with iodoacetamide. S-nitrosylated cysteine residues were selectively reduced with sodium ascorbate and derivatized with a Cys-Tag (CPT) reagent. After tryptic digestion, CPT-labeled peptides were enriched using immobilized metal affinity chromatography (IMAC) and analyzed by DIA LC–MS/MS on the same Orbitrap Astral platform. Raw data were processed using Spectronaut, and S-nitrosylation sites were quantified by intensity-based label-free quantification. PTM sites with at least three valid quantitative values in either group were retained. Differential expression analysis was performed using the limma package in R (|log2 fold change| > 1, P < 0.05), and functional enrichment analysis was conducted using the clusterProfiler R package.

### 2.4 Metabolomics

Metabolites were extracted and analyzed by LC-MS/MS as previously described [12]. Internal standards were used to monitor retention-time stability. Pooled QC samples were prepared by combining equal aliquots from all study samples and were analyzed at regular intervals throughout the analytical sequence to assess analytical reproducibility. For the C18 column, the retention-time drift of the internal standards was required to be ≤ 0.3 min. For the amide column, the retention time of the internal standards was required to fall within the predefined acceptance window and not exceed 9.6 min. Metabolites with an RSD >30% in pooled QC samples were excluded from downstream analysis. Differential expression analysis was performed using the limma package in R (|log2 fold change| > 1, P < 0.05). Functional enrichment analysis was performed using MetaboAnalyst.

### 2.5 Cardiomyocyte isolation

Cardiomyocytes were isolated from CTL, HFpEF, and HFpEF + Na-βHB mice using a modified Langendorff-free method for further evaluation as previously described [13,14]. Briefly, hearts were perfused with EDTA buffer (130 mmol/L NaCl, 5 mmol/L KCl, 0.5 mmol/L NaH_2_PO_4_, 10 mmol/L HEPES, 10 mmol/L glucose, 10 mmol/L 2,3-butanedione monoxime, 10 mmol/L taurine, and 5 mmol/L EDTA, pH 7.8) for 3 min, followed by calcium-free perfusion buffer (same composition with EDTA buffer, except that EDTA was omitted and 1 mmol/L MgCl_2_ was added, pH 7.8) for 3 minutes, and then enzymatically digested at 37°C for approximately 15 minutes with perfusion buffer containing 0.5 μg/mL collagenase II (Cat. No. LS004176, Worthington), 0.5 μg/mL collagenase IV (Cat. No. LS004188, Worthington), and 0.05 μg/mL protease XIV (Cat. No. P5147, Sigma). Digestion time was adjusted according to heart size. Ventricular tissue was then gently dissociated and passed through a 250 μm mesh, and cardiomyocytes were separated from non-cardiomyocytes by gravity sedimentation. After isolation, cardiomyocytes designed for stable isotope tracing were immediately subjected to the tracer incubation, whereas cells used for metabolomics and NADH/NAD^+^ analyses were processed or snap-frozen according to the respective assay protocol.

### 2.6 Immunoblotting

Whole ventricular heart tissue lysates were prepared in freshly made RIPA buffer containing 150 mmol/L NaCl, 50 mmol/L Tris-HCl, 1% Triton X-100 (Cat. No. 9002-93-1, Sigma-Aldrich), 0.5% sodium deoxycholate, and 0.1% SDS. The buffer was supplemented with 1% orthovanadate (Cat. No. S6508-50g, Merck), 1% okadaic acid sodium salt (Cat. No. O-5857-1mg, LC Laboratories), 0.8% phenylmethylsulfonyl fluoride (Cat. No. 36978, Thermo Fisher Scientific), and 1.2% protease inhibitor mixture containing antipain, aprotinin, chymostatin, leupeptin, pepstatin, and trypsin inhibitor (Applichem). Protein concentrations were determined using the Bradford assay. Equal amounts of protein were mixed with 3× SDS sample buffer containing 25.5% glycerol, 6% SDS, 188 mmol/L Tris-HCl (pH 6.8), 60 mmol/L DTT, and 0.006% bromophenol blue. Proteins were separated by SDS-PAGE and transferred to nitrocellulose membranes using a wet-transfer system (Bio-Rad, Hercules, CA, USA). Membranes were blocked with 1× ROTI Block (Cat. No. A151.3, Carl Roth) and incubated overnight at 4°C with primary antibodies diluted in blocking buffer. After washing, membranes were incubated for 2 hours at room temperature with horseradish peroxidase-conjugated secondary antibodies (1:10,000; Cat. No. 401393 or 401253, Merck Millipore). Signals were developed using Clarity Western ECL Substrate (Cat. No. 1705061, Bio-Rad) and acquired with a ChemiDoc Imaging System. Band intensities were quantified using the Gel Analyzer function in ImageJ after background subtraction, normalized to the loading control, and expressed as fold change relative to the reference sample unless otherwise stated. Antibodies used for Western blot are: ACAT1 antibody was from Creative Biolabs (Cat. No. MOR-0031, Creative Biolabs, Belgium), BDH1 and OXCT1 antibodies were from Abcam (Cat. No. ab193156 and Cat. No. ab224250, Abcam, Darmstadt), Vinculin monoclonal antibody was from Thermo Fisher Scientific (Cat. No. MA5-11690, Invitrogen, Harz, Germany).

### 2.7 Stable isotope tracing in isolated adult cardiomyocytes

Freshly isolated adult mouse cardiomyocytes from CTL, HFpEF, and HFpEF + Na-βHB mice were resuspended in a buffer containing 113 mmol/L NaCl, 4.7 mmol/L KCl, 0.6 mmol/L KH_2_PO_4_, 0.6 mmol/L Na_2_HPO_4_, 1.2 mmol/L MgSO_4_, 12 mmol/L NaHCO_3_, 10 mmol/L KHCO_3_, 10 mmol/L HEPES, 30 mmol/L taurine, 5.5 mmol/L glucose, 10 mmol/L 2,3-butanedione monoxime, and 5 mg/mL bovine serum albumin (BSA). Extracellular Ca^2+^ was gradually reintroduced in a stepwise manner from 0.1 mmol/L to a final concentration of 1.2 mmol/L. Cardiomyocytes were subsequently washed with substrate-free Krebs–HEPES buffer (135 mmol/L NaCl, 4.7 mmol/L KCl, 1.2 mmol/L KH_2_PO_4_, 1.2 mmol/L MgSO_4_, 1.2 mmol/L CaCl_2_, 10 mmol/L HEPES, pH 7.4). Cells were then incubated for 1 hour at 37°C in Krebs–HEPES buffer containing glucose, palmitate, and 3-hydroxybutyrate at concentrations of 1, 0.25, and 0.1 mmol/L, respectively. In each parallel condition, only one substrate was supplied in uniformly ^13^C-labeled form [U-^13^C_6_]glucose (Cat. No. 389374, Sigma-Aldrich, Germany), fatty acid free BSA-conjugated [U-^13^C_16_]palmitate (Cat. No. 56599-85-0, Sigma-Aldrich, Germany), or [U-^13^C_4_]3-hydroxybutyrate (Cat. No. 287111-43-7, MedChemExpress, USA), while the other two substrates remained unlabeled. Acetyl-CoA isotopologue enrichment was subsequently quantified by LC–MS/MS as a measure of substrate-specific carbon contribution. After incubation, cells were washed with ice-cold PBS, and harvested in 1 mL of ice-cold 85% methanol. Samples were sonicated on ice for 3 min, briefly vortexed, incubated on ice for 20 minutes, and sonicated again for 2 minutes. Samples were then centrifuged at 14,000 rpm for 10 minutes at 4°C, and the supernatants were analyzed by LC–MS/MS. Isotopologue distributions were corrected for natural ^13^C abundance, and the contribution of each substrate to acetyl-CoA was calculated as the percentage of labeled acetyl-CoA relative to the total acetyl-CoA pool.

### 2.8 Mitochondrial respiration assay

Mitochondrial oxygen consumption rate (OCR) was measured in freshly prepared left-ventricular tissue pieces using a Seahorse XFe24 analyzer, as previously described with minor modifications [13]. After heart excision, the great vessels, atria, and right ventricle were removed, and LV tissue was cut into pieces of comparable size and weight and placed in Seahorse islet-capture plates. Tissue pieces were maintained in Seahorse XF DMEM assay medium supplemented with 10 mmol/L glucose, 2 mmol/L glutamine, and 1 mmol/L pyruvate and equilibrated for 1 hour at 37°C in a non-CO_2_ incubator. Baseline OCR was recorded, followed by sequential injection of oligomycin (1.25 μmol/L), FCCP (3 μmol/L), and rotenone/antimycin A (3 and 10 μmol/L, respectively). Basal mitochondrial respiration was calculated by subtracting non-mitochondrial OCR measured after rotenone/antimycin A from baseline OCR. ATP-linked respiration was calculated as the oligomycin-sensitive decrease in OCR. OCR values were normalized to tissue weight.

### 2.9 MitoSOX measurement

Freshly isolated cardiomyocytes were incubated with MitoSOX™ Red reagent (1:5000 in PBS with Ca^2+^/Mg^2+^) for 30 minutes at 37°C, and fluorescence was quantified by flow cytometry analysis (BD FACSCanto™ II). Viable single cells were gated based on FSC/SSC characteristics. Data were analyzed using FlowJo V10.

### 2.10 NADH/NAD^+^assay

The assay was performed in freshly isolated and snap-frozen cardiomyocytes with a commercially available NADH/NAD^+^ assay kit from Abcam (Berlin, Germany, Cat. No. ab176723) according to the manufacturer's protocol as described previously [13].

### 2.11 Statistical analysis

All data are presented as the mean ± SD. One-way analysis of variance (ANOVA) followed by Tukey's multiple comparisons test was used. Details are presented on each figure legend. All analyses were performed using GraphPad PRISM data analysis software (version 10.2; GraphPad Software).

## 3 Results

### 3.1 Cardiometabolic HFpEF shows modest proteomic remodeling but marked alterations in mitochondrial substrate handling

**Fig. 1:**
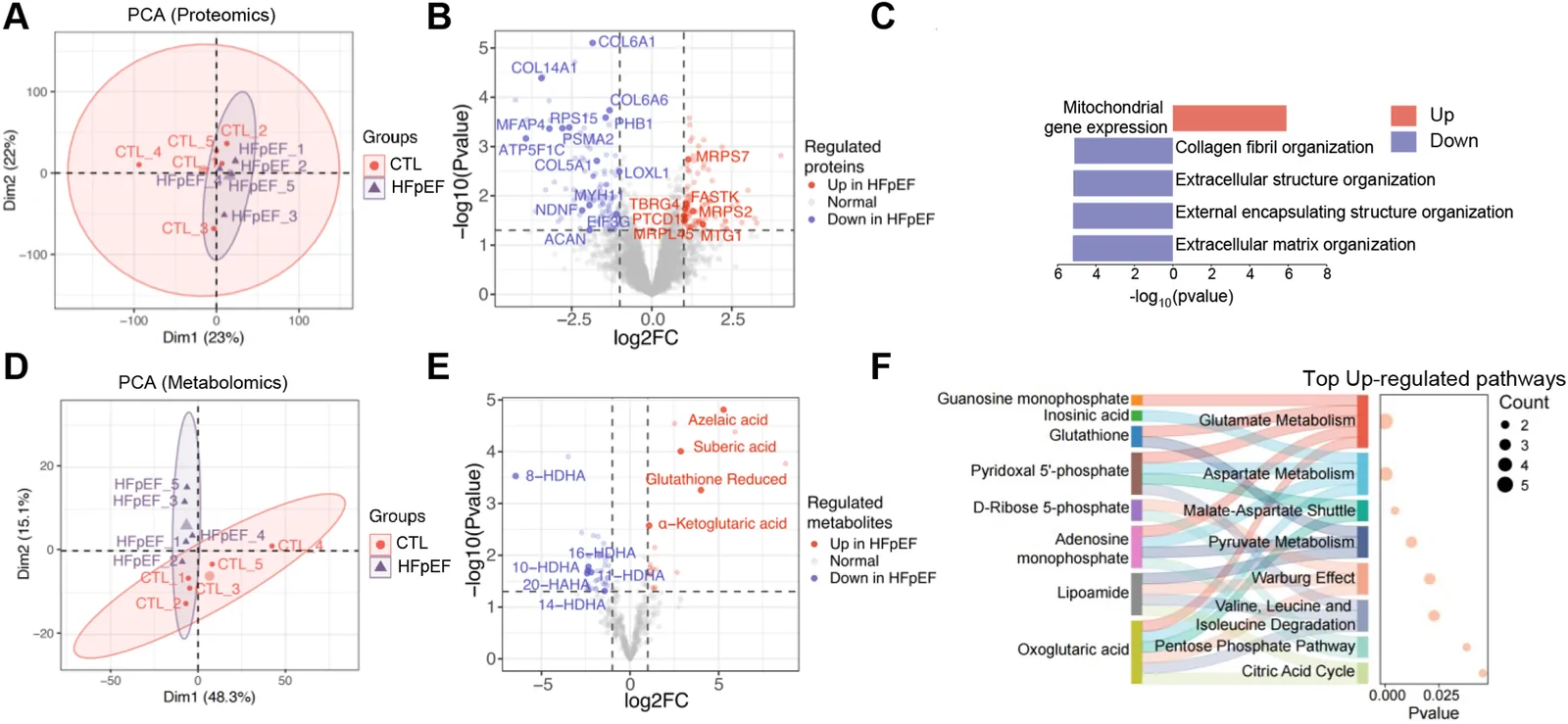
**Metabolic HFpEF shows modest proteomic remodeling but metabolic evidence of altered mitochondrial substrate handling. A–B.** Principal component analysis (PCA) (A) and volcano plot (B) of cardiac proteomes from mice under normal diet (CTL) or treated with high-fat diet and l-NAME (HFpEF). **C.** Bar plot displaying biological pathways enriched among differential proteins between CTL and HFpEF groups. **D–E.** PCA (D) and volcano plot (E) of cardiac metabolomes from the CTL and HFpEF groups. **F.** Integrated bubble plot and Sankey diagram depicting metabolic pathways enriched in HFpEF. Metabolites driving pathway enrichment are highlighted on the left.

Using the two-hit HFD/l-NAME model of metabolic HFpEF, we found modest global proteomic alterations (Fig. 1A, Table S1). Differentially expressed proteins were enriched in cytoskeletal/contractile components (MYH11, TAGLN, CNN1, VIM) and extracellular matrix (COL5A1, COL6A1, COL6A6, COL14A1) (Fig. 1B and C, Table S1), indicating coordinated structural remodeling despite limited abundance-level proteome reprogramming. In contrast, metabolomic profiling revealed a more prominent shift in pathways linked to mitochondrial substrate handling (Fig. 1D and E, Table S2). TCA-cycle remodeling was selective, with increased α-ketoglutarate, unchanged citrate, and more modest changes in succinate, fumarate, and malate. In parallel, the dicarboxylic acids, azelaic acid and suberic acid were markedly elevated, consistent with altered fatty-acid handling and possible engagement of ω-oxidation pathways. Redox-associated metabolites, including reduced glutathione, were also increased, reflecting activation of compensatory antioxidant mechanisms. In contrast, multiple ω-3 polyunsaturated fatty acid–derived oxylipins were decreased, pointing to impaired protective lipid signaling and altered fatty acid handling (Fig. 1E and F). However, in human HFpEF, some TCA-cycle intermediates such as succinate and fumarate are reduced. Likewise, fatty acid-related metabolites such as medium- and long-chain acylcarnitines, as well as fatty acid oxidation proteins and genes, are generally decreased in human HFpEF [[15], [16], [17]]. Therefore, the murine and human findings may represent distinct patterns of mitochondrial substrate handling in HFpEF. The murine model reflects a state of substrate overload with redox-constrained oxidative flux, whereas human HFpEF myocardium may represent a more advanced or differently organized state characterized by reduced oxidative organization and impaired substrate utilization capacity.

Taken together, these data identify a metabolite pattern in the HFD/l-NAME model that is consistent with altered oxidative metabolism and mitochondrial redox imbalance. Although steady-state metabolite levels do not allow direct inference of pathway flux, the pattern is consistent with altered mitochondrial substrate handling and redox-metabolic remodeling in this experimental setting.

### 3.2 S-nitrosylation remodeling identifies redox-sensitive metabolic control nodes in the HFD/l-NAME model

**Fig. 2:**
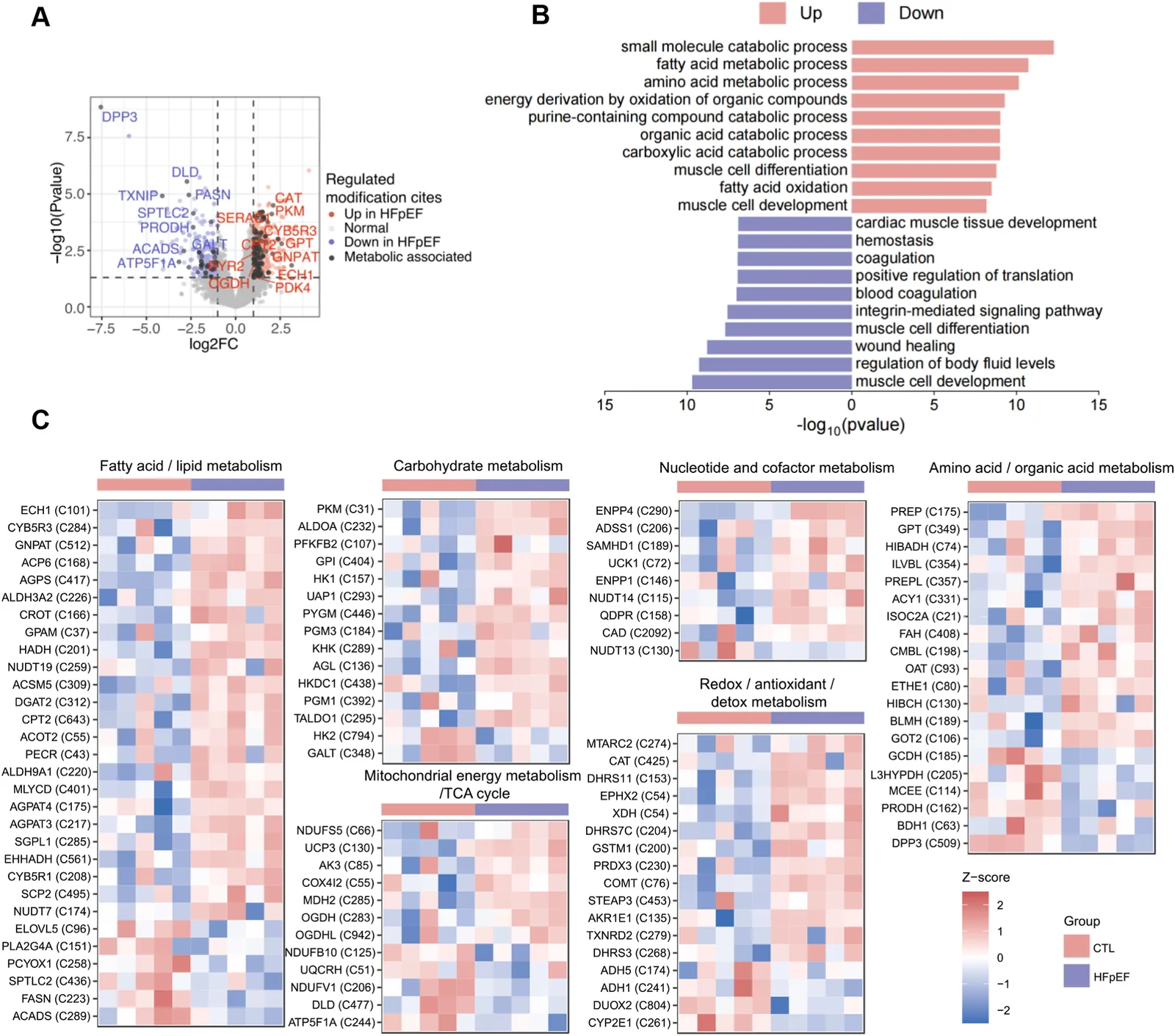
**S-nitrosylation is remodeled in HFpEF. A.** Volcano plot identifying differentially S-nitrosylated cysteines in HFpEF compared to CTL hearts. **B.** Bar plot displaying biological pathways enriched among proteins harboring differentially S-nitrosylated cysteines between CTL and HFpEF groups. **C.** Heatmap displaying Z-scores of differentially S-nitrosylated cysteines of key metabolic proteins across major metabolic pathways.

To explore how metabolic remodeling might be regulated in this experimental setting, we examined cysteine S-nitrosylation as a candidate redox-sensitive post-translational mechanism. Because nitric oxide-related signaling can modulate protein function, enzymatic activity, and substrate utilization through reversible cysteine modification, we asked whether the HFD/l-NAME model exhibits coordinated S-nitrosylation changes across metabolic pathways. Indeed, S-nitrosylation remodeling in HFpEF was significantly altered (Fig. 2A, Table S3). Sites with increased S-nitrosylation were associated with proteins involved in mitochondrial redox defenses and metabolic control, including enzymes regulating fatty acid utilization and oxidative flux such as PDK4, MLYCD, CPT2, and OGDH, as well as contractile and Ca^2+^ handling proteins including RYR2 and SERCA2 (Fig. 2A–Table S3). Conversely, sites with decreased S-nitrosylation affected complementary modules of contractile structure and fuel handling, consistent with dynamic, site-specific redox regulation of mitochondrial substrate handling (Fig. 2A). In agreement with these findings, Gene Ontology analysis of the differentially S-nitrosylated proteins identified a strong enrichment for pathways governing catabolic metabolism and energy turnover, including small-molecule catabolism, fatty acid metabolism and oxidation, amino-acid metabolism, organic/carboxylic-acid catabolism, and energy derivation by oxidation of organic compounds (Fig. 2B). Additional enrichment in muscle cell differentiation/development suggests that this metabolic rewiring occurs in parallel with remodeling of cardiac structural programs. Notably, these enrichments were centered on cardiac metabolic processes, rather than broad transcriptional pathways, supporting the concept that HFpEF is characterized by a post-translationally regulated metabolic phenotype. Next, we mapped the differential S-nitrosylation sites of key metabolic proteins across major metabolic modules (Fig. 2C). Distinct and coordinated remodeling was evident in fatty acid/lipid metabolism, carbohydrate metabolism, mitochondrial energy metabolism/TCA cycle, nucleotide and cofactor metabolism, amino acid/organic acid metabolism, and redox/antioxidant/detox pathways. Across these modules, S-nitrosylation changes indicated selective remodeling of metabolic control nodes instead of uniform activation or suppression of entire pathways. Together with the metabolomic alterations described above, these findings are consistent with redox-sensitive post-translational regulation contributing to altered mitochondrial substrate handling in the HFD/l-NAME model.

### 3.3 Beta-hydroxybutyrate relieves redox-constrained oxidative metabolism and improves HFpEF phenotypes

To test whether the redox-metabolic state identified in the HFD/l-NAME model could be functionally modified, we examined the effects of shifting substrate availability toward ketone metabolism. Because beta-hydroxybutyrate (β-OHB) can be metabolized to acetyl-CoA through the ketolytic pathway and enter the TCA cycle independently of carnitine-dependent long-chain fatty acid transport [18], we reasoned that shifting substrate availability toward ketone metabolism might reduce fatty acid-associated redox burden and modify mitochondrial substrate use.

**Fig. 3:**
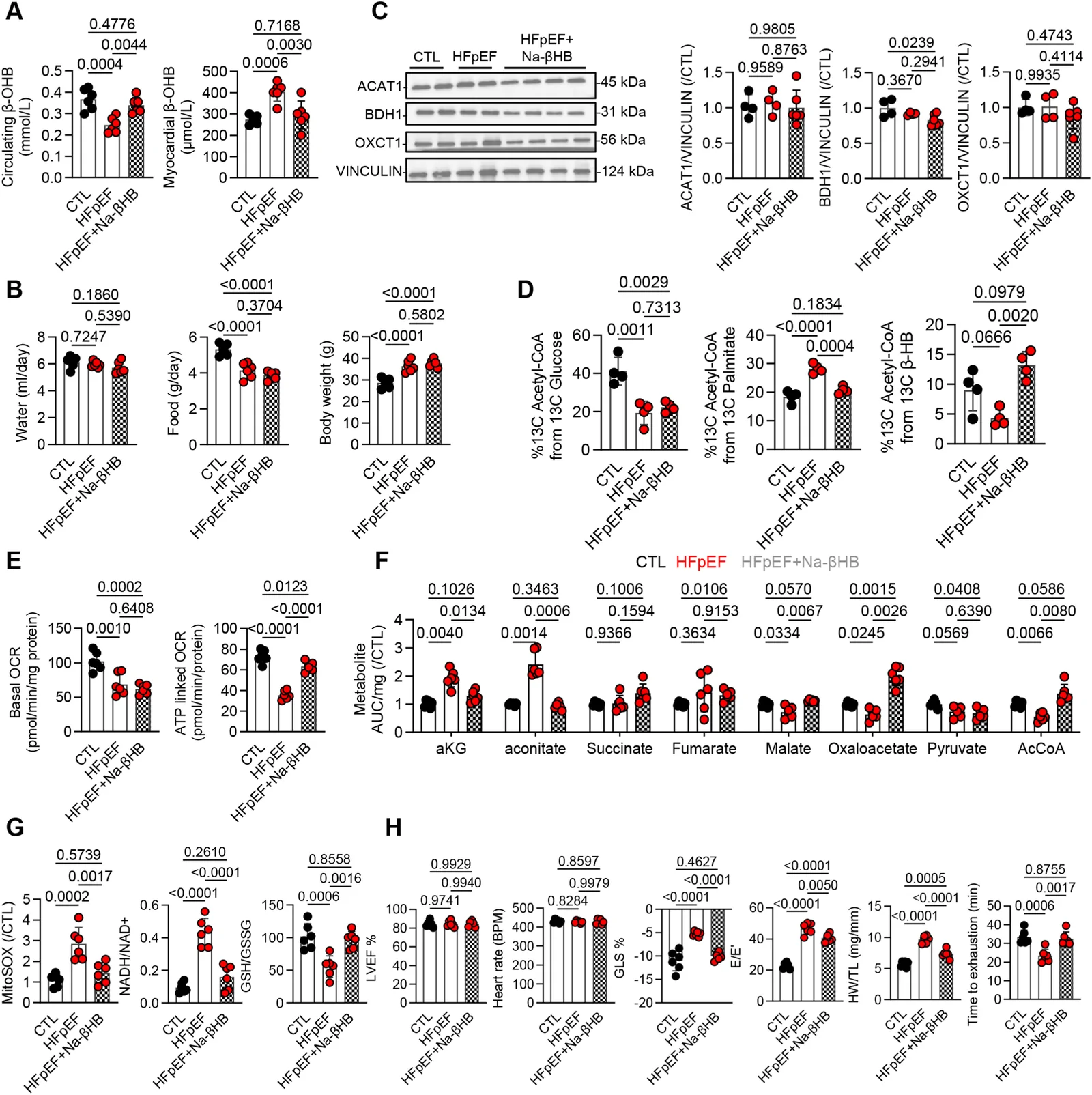
**Na-βHB supplementation modifies substrate handling and improves redox-metabolic and functional phenotypes in HFpEF. A.** Circulating β-OHB (left) and myocardial β-OHB (right) levels in CTL, HFpEF, and Na-βHB-supplemented HFpEF mice. **B.** Water intake (left), food intake (middle), or body weight (right) of mice as in panel A. **C.** Immunoblot analysis and quantification of cardiac ACAT1, BDH1, and OXCT1 in cardiac ventricular tissue from mice as in panel A. **D.** Fractional contribution of ^13^C-glucose, ^13^C-palmitate, and ^13^C-βHB-derived carbon to acetyl-CoA in isolated cardiomyocytes from mice as in panel A. **E.** Basal and oligomycin-sensitive respiration measured in ventricular cardiac tissue isolated from mice as in panel A. **F.** TCA-cycle metabolites, pyruvate, and acetyl-CoA in isolated cardiomyocytes from mice as in panel A. **G.** MitoSOX fluorescence intensity (left), NADH/NAD^+^ ratio (middle) and GSH/GSSG ratio (right) in isolated cardiomyocytes from mice as in panel A. **H.** Percent left ventricular ejection fraction (LVEF), heart rate, left ventricular global longitudinal strain (GLS), ratio between mitral E wave and E′ wave (E/E′), ratio between heart weight and tibia length (HW/TL), and running time to exhaustion in mice as in panel A. Results are presented as mean ± SD. One-way ANOVA, Tukey's multiple-comparisons test (A–H). n = 4-6/group.

Na-βHB supplementation had a mild effect on circulating β-OHB levels while reducing cardiac β-OHB levels toward those observed in CTL mice (Fig. 3A). These changes occurred without significant differences in water intake, food intake, or body weight between untreated and Na-βHB-supplemented HFpEF mice (Fig. 3B). Because reduced myocardial expression of ketolytic enzymes has been reported in experimental and human HFpEF [16,[19], [20], [21]], we assessed the abundance of BDH1, which converts β-hydroxybutyrate to acetoacetate, OXCT1, which activates acetoacetate to acetoacetyl-CoA, and ACAT1, which generates acetyl-CoA from acetoacetyl-CoA. None of these enzymes was reduced in HFpEF hearts compared with CTL hearts. Na-βHB supplementation did not alter ACAT1 or OXCT1 abundance but modestly reduced BDH1 protein levels (Fig. 3C), indicating that the increased β-OHB-derived contribution to acetyl-CoA was not explained by upregulation of the canonical ketolytic machinery. Importantly, stable-isotope tracing in isolated cardiomyocytes further demonstrated altered substrate contribution to acetyl-CoA production. Compared with CTL cardiomyocytes, HFpEF cardiomyocytes showed reduced glucose-derived and increased palmitate-derived acetyl-CoA labeling, whereas β-OHB-derived labeling was not significantly different. In cardiomyocytes from Na-βHB-supplemented HFpEF mice, the elevated palmitate-derived contribution was reduced and the β-OHB-derived contribution was increased, while the reduced glucose-derived contribution remained unchanged (Fig. 3D). These findings indicate that HFpEF shifts mitochondrial substrate utilization away from glucose toward fatty acids with a trend to reduce ketone utilization, whereas Na-βHB supplementation redirects acetyl-CoA production without restoring glucose utilization. In parallel, analysis of freshly prepared LV tissue showed that oligomycin-sensitive respiration was increased in Na-βHB-supplemented HFpEF mice compared with untreated HFpEF mice, whereas basal respiration remained reduced and was not significantly different between the two HFpEF groups (Fig. 3E). Na-βHB supplementation also selectively remodeled several TCA-cycle-associated metabolites, including α-ketoglutarate, aconitate, malate, and oxaloacetate, whereas succinate and fumarate remained unchanged (Fig. 3F). Pyruvate differed mildly, but acetyl-CoA was elevated after Na-βHB supplementation (Fig. 3F). In parallel, Na-βHB attenuated redox stress, as indicated by reduced MitoSOX fluorescence, a lower NADH/NAD^+^ ratio, and restoration of the GSH/GSSG ratio (Fig. 3G). These metabolic and redox changes were associated with improved cardiac performance characterized by better global longitudinal strain (GLS) and lower E/E′, together with reduced heart weight/tibia length and improved exercise capacity, whereas neither LVEF nor heart rate differed among the three groups, consistent with the HFpEF phenotype (Fig. 3H).

Together, these findings indicate that long-term Na-βHB supplementation is associated with coordinated metabolic, redox, and functional effects in the HFD/l-NAME model. Because supplementation was initiated at the onset of disease induction, these effects may reflect long-term metabolic adaptation or prevention of maladaptive substrate selection rather than reversal of an established metabolic defect. Although these data do not establish the precise metabolic bottleneck engaged by Na-βHB, they support the concept that altering mitochondrial substrate use can partially rescue mitochondrial and functional abnormalities in this experimental setting.

## 4 Discussion

In this study, we define a redox-sensitive mitochondrial metabolic state in the HFD/l-NAME murine model of metabolic HFpEF. Although global changes in the cardiac proteome were relatively modest, integrated metabolomic and nitrosoproteomic profiling revealed altered mitochondrial substrate handling, redox stress, and structured S-nitrosylation remodeling of key control nodes. Within this experimental context, these data support the interpretation that mitochondrial dysfunction is associated less with broad loss of mitochondrial proteins and more with regulatory remodeling at the level of protein modification and metabolic organization.

Several findings support this interpretation. First, the steady-state metabolomic profile showed non-uniform remodeling of TCA-cycle intermediates, increased dicarboxylic acids, and altered redox-associated metabolites. Although these measurements do not establish pathway directionality or flux, together with the reduced respiratory function observed in ventricle tissue, they support the presence of altered substrate handling and redox imbalance in the HFD/l-NAME model. Second, our S-nitrosylation proteomics indicate that this metabolic state is accompanied by highly structured and bidirectional redox-sensitive remodeling, rather than a nonspecific increase in oxidative damage. Differential S-nitrosylation was enriched in multiple metabolic modules, including fatty acid/lipid metabolism, carbohydrate metabolism, mitochondrial energy metabolism, amino acid/organic acid metabolism, nucleotide/cofactor metabolism, and redox defense pathways. In parallel, S-nitrosylation changes were also present in proteins involved in contractile and Ca^2+^ handling pathways. This organization is notable because it suggests that metabolic and mechanical remodeling may be linked through shared redox-sensitive regulatory processes. At the same time, our data do not establish the functional directionality of individual S-nitrosylation events, and site-specific validation will be required to determine which modified proteins act as causal control nodes in this model.

An important implication of the present study is that the HFD/l-NAME system should be interpreted as an experimental platform rather than a faithful metabolic surrogate of human HFpEF. Although it is among the most widely accepted and commonly used animal models of HFpEF, it captures only selected aspects of human HFpEF, which is highly heterogeneous and exhibits considerable diversity across populations shaped by comorbidity burden, age, sex, and disease duration in ways that are difficult to reproduce in mice [22]. Comparison with human myocardial studies suggests that its metabolic organization may differ in important respects from that observed in human HFpEF. In our study, remodeling of TCA-cycle intermediates together with increased dicarboxylic acids suggests excessive fatty acid-derived substrate pressure with downstream oxidative constraint in HFpEF mice. By contrast, Hahn et al. reported reduced medium- and long-chain acylcarnitines, increased pyruvate, lower succinate/fumarate, and accumulation of BCAAs with reduced downstream catabolites in human HFpEF myocardium, a pattern more consistent with impaired substrate utilization capacity and reduced oxidative organization [15]. Thus, while both datasets indicate altered myocardial metabolic organization, the murine model may reflect a state of substrate overload and redox backpressure, whereas human HFpEF may exhibit a broader failure of coordinated fuel utilization. We therefore view the present findings not as evidence that the murine HFD/l-NAME model recapitulates the human metabolic syndrome, but rather as evidence that this widely used model engages a distinct and experimentally tractable redox-metabolic program under cardiometabolic stress. In this sense, the divergence from human HFpEF is not merely a limitation but also informative, because it clarifies what this model can and cannot be used to study.

The use of ketones as a therapeutic strategy in HFpEF has also been reported by other groups, although the mechanistic emphasis and outcomes have differed across studies. The recent study by Sun et al. (2025) showed that unlike in HFrEF, cardiac BDH1 and SCOT/OXCT1 expression was decreased, together with impaired myocardial ketone oxidation in the HFD/l-NAME model, suggesting that ketone metabolic remodeling is less prominent in HFpEF. However, this does not exclude the possibility that exogenous ketone supplementation may exert beneficial effects through acute bioenergetic, hemodynamic, or extracardiac mechanisms in selected HFpEF phenotypes [19]. By contrast, in our HFD/l-NAME model, cardiac ACAT1 and OXCT1 protein abundance remained unchanged, whereas BDH1 protein abundance was modestly reduced after Na-βHB supplementation. Nevertheless, stable isotope tracing demonstrated an increased contribution of β-OHB-derived carbon to acetyl-CoA despite the reduction in BDH1 abundance, suggesting that the treatment-associated metabolic adaptation cannot be explained simply by changes in the abundance of canonical ketolytic enzymes. In the HFD/l-NAME model, Liao et al. showed that β-OHB improved diastolic dysfunction and cardiac remodeling, and interpreted these effects mainly in the context of increased cardiac Treg cells and suppression of the NOX2/GSK-3β pathway, thus emphasizing an anti-inflammatory and immunomodulatory mechanism [23]. Deng et al. likewise reported that increasing β-OHB availability ameliorated HFpEF, and linked this benefit primarily to suppression of a mitochondrial hyperacetylation-NLRP3 inflammasome circuit [24]. Our study differs from these prior reports in both question and mechanistic framework. Rather than focusing primarily on inflammation or immune regulation, we examined β-OHB supplementation in the context of mitochondrial substrate handling and redox homeostasis. Na-βHB supplementation selectively increased the oligomycin-sensitive component of respiration, whereas basal respiration was not significantly restored. Because these measurements were obtained in *ex vivo* ventricular tissue pieces containing multiple cardiac cell types, oligomycin-sensitive OCR should be interpreted as the component of tissue respiration coupled to ATP synthesis under the assay conditions, rather than as a direct measure of cardiomyocyte ATP demand, contractile workload, or energetic efficiency. Considered together with the substrate-tracing results obtained in isolated cardiomyocytes and the accompanying changes in TCA-cycle-associated metabolites and redox indices, this finding is consistent with coordinated remodeling of myocardial metabolism and redox homeostasis. Because Na-βHB supplementation was initiated at the onset of the HFD/l-NAME protocol and maintained throughout disease development, the observed increase in β-OHB-derived acetyl-CoA labeling is unlikely to reflect restoration of ketolytic enzyme expression. Rather, it is more consistent with long-term metabolic adaptation to sustained substrate availability, resulting in a greater contribution of β-OHB-derived carbon to acetyl-CoA despite the absence of increased canonical ketolytic enzyme abundance. The differences among studies likely reflect differences in HFpEF models, treatment modality, exposure duration, and the specific metabolic defect being interrogated. Importantly, our findings do not contradict prior reports of impaired ketolytic remodeling in HFpEF. Instead, they suggest that early and continuous Na-βHB supplementation can remodel mitochondrial substrate utilization without requiring increased expression of canonical ketolytic enzymes. This places ketone supplementation within a broader mechanistic context in which substrate redistribution, mitochondrial acetylation, redox regulation and anti-inflammatory signaling may all intersect with redox-sensitive control of mitochondrial metabolism.

Our study has several limitations. We examined a specific cardiometabolic HFpEF model, and the mechanisms identified here should not be generalized to other experimental HFpEF phenotypes. In addition, the exclusive use of male mice limits the generalizability of our findings, particularly given the higher prevalence of HFpEF in women [25]. Whether the metabolic and S-nitrosylation remodeling identified here is sex dependent remains to be determined. Although our findings implicate S-nitrosylation as a candidate regulatory layer associated with metabolic remodeling, site-specific functional studies are required to establish causal links between individual S-nitrosylation events and altered metabolic flux. Physical activity was not monitored, and treatment-related changes in spontaneous activity may therefore have contributed to the observed metabolic or cardiac effects. In addition, *in vivo* substrate flux, ATP production, and energetic efficiency were not directly measured. Finally, S-nitrosylation proteomics was not performed in Na-βHB-supplemented hearts. Therefore, whether Na-βHB modifies the cardiac S-nitrosylation landscape remains unresolved.

Despite these limitations, our findings clarify the metabolic and redox features captured by the HFD/l-NAME model and define the boundaries of its relevance to human HFpEF. Within this experimental context, Na-βHB supplementation was associated with partial modification of the substrate-handling, redox, and functional phenotype. These results support the use of the HFD/l-NAME model as a platform for studying high-fat-induced cardiometabolic stress, while emphasizing that its metabolic organization should not be assumed to reproduce that of human HFpEF.

## CRediT authorship contribution statement

**Huihui Li:** Conceptualization, Data curation, Formal analysis, Investigation, Methodology, Visualization, Writing – original draft, Writing – review & editing. **Hanyu Cui:** Investigation, Methodology, Writing – review & editing. **Daiyu Wang:** Investigation, Methodology. **Florian Leuschner:** Writing – review & editing. **Joerg Heineke:** Writing – review & editing. **Jiong Hu:** Funding acquisition, Resources, Supervision, Writing – review & editing. **Sofia-Iris Bibli:** Conceptualization, Funding acquisition, Supervision, Writing – original draft, Writing – review & editing.

## Ethics declaration

This study was conducted in accordance with the ARRIVE (Animal Research: Reporting of In Vivo Experiments) guidelines. This study was approved by the University Animal Care Committee and the Federal Authority for Animal Research at Tongji Medical College.

## Disclosures

None.

## Funding

This work was supported by the 10.13039/501100001659Deutsche Forschungsgemeinschaft (CRC1531/1 Project A02 to S.-I.B., B07 to F.L., Project ID 456687919; CRC1366/2 Project B01 to S.-I.B., Project A06 to J.H., Project ID 39404578; The Emmy Noether Programme BI 2163/1-2 to S.-I.B.); The Natural Science Foundation of China, Project IDs 82070501, 82271479 and 32350021 to J.H.; The Natural Science Foundation of China, Project ID 82500447 to H.L; The China Postdoctoral Science Foundation, Project IDs 2024M761043, 2025T073HB to H.L.

## Declaration of competing interests

The authors declare no conflicts of interest.

## Data Availability

Data will be made available on request.

## References

[1] Fayyaz A.U., Eltony M., Prokop L.J., Koepp K.E., Borlaug B.A., Dasari S. (2025). Pathophysiological insights into HFpEF from studies of human cardiac tissue. Nat. Rev. Cardiol..

[2] Mericskay M., Zuurbier C.J., Heather L.C., Karlstaedt A., Inserte J., Bertrand L. (2025). Cardiac intermediary metabolism in heart failure: substrate use, signalling roles and therapeutic targets. Nat. Rev. Cardiol..

[3] Daou D., Tong D., Schiattarella G.G., Gillette T.G., Hill J.A. (2025). What is cardiometabolic HFpEF and how can we study it preclinically?. JACC, Basic Transl. Sci..

[4] Schiattarella G.G., Altamirano F., Tong D., French K.M., Villalobos E., Kim S.Y. (2019). Nitrosative stress drives heart failure with preserved ejection fraction. Nature.

[5] Kumar A.A., Kelly D.P., Chirinos J.A. (2019). Mitochondrial dysfunction in heart failure with preserved ejection fraction. Circulation.

[6] Liu X., Zhang Y., Deng Y., Yang L., Ou W., Xie M. (2022). Mitochondrial protein hyperacetylation underpins heart failure with preserved ejection fraction in mice. J. Mol. Cell. Cardiol..

[7] Tong D., Schiattarella G.G., Jiang N., Altamirano F., Szweda P.A., Elnwasany A. (2021). NAD^+^ repletion reverses heart failure with preserved ejection fraction. Circ. Res..

[8] Peoples J.N., Saraf A., Ghazal N., Pham T.T., Kwong J.Q. (2019). Mitochondrial dysfunction and oxidative stress in heart disease. Exp. Mol. Med..

[9] Carlstrom M., Weitzberg E., Lundberg J.O. (2024). Nitric oxide signaling and regulation in the cardiovascular system: recent advances. Pharmacol. Rev..

[10] Boehm M., Lawrie A., Wilhelm J., Ghofrani H.A., Grimminger F., Weissmann N. (2017). Maintained right ventricular pressure overload induces ventricular-arterial decoupling in mice. Exp. Physiol..

[11] Zampas P., Li Z., Katsouda A., Varela A., Psarras S., Davos C.H. (2025). Protective role of 3-mercaptopyruvate sulfurtransferase (MPST) in the development of metabolic syndrome and vascular inflammation. Pharmacol. Res..

[12] Drekolia M.K., Mettner J., Wang D., Delgado Lagos F., Koch C., Hecker D. (2026). Cystine import and oxidative catabolism fuel vascular growth and repair via nutrient-responsive histone acetylation. Cell Metab..

[13] Drekolia M.K., Karantanou C., Wittig I., Li Y., Fuhrmann D.C., Brune B. (2024). Loss of cardiac mitochondrial complex I persulfidation impairs NAD^+^ homeostasis in aging. Redox Biol..

[14] Ackers-Johnson M., Li P.Y., Holmes A.P., O'Brien S.M., Pavlovic D., Foo R.S. (2016). A simplified, langendorff-free method for concomitant isolation of viable cardiac myocytes and nonmyocytes from the adult mouse heart. Circ. Res..

[15] Hahn V.S., Petucci C., Kim M.S., Bedi K.C., Wang H., Mishra S. (2023). Myocardial metabolomics of human heart failure with preserved ejection fraction. Circulation.

[16] Jani V.P., Yoo E.J., Binek A., Guo A., Kim J.S., Aguilan J. (2025). Myocardial proteome in human heart failure with preserved ejection fraction. J. Am. Heart Assoc..

[17] O'Sullivan J.F., Li M., Koay Y.C., Wang X.S., Guglielmi G., Marques F.Z. (2024). Cardiac substrate utilization and relationship to invasive exercise hemodynamic parameters in HFpEF. JACC, Basic Transl. Sci..

[18] Newman J.C., Verdin E. (2017). Beta-hydroxybutyrate: a signaling metabolite. Annu. Rev. Nutr..

[19] Sun Q., Wagg C.S., Wong N., Wei K., Ketema E.B., Zhang L. (2025). Alterations of myocardial ketone metabolism in heart failure with preserved ejection fraction (HFpEF). ESC Heart Fail..

[20] Koay Y.C., McIntosh B., Ng Y.H., Cao Y., Wang X.S., Han Y. (2025). The heart has intrinsic ketogenic capacity that mediates NAD(+) therapy in HFpEF. Circ. Res..

[21] Sun Q., Karwi Q.G., Wong N., Lopaschuk G.D. (2024). Advances in myocardial energy metabolism: metabolic remodelling in heart failure and beyond. Cardiovasc. Res..

[22] Withaar C., Lam C.S.P., Schiattarella G.G., de Boer R.A., Meems L.M.G. (2021). Heart failure with preserved ejection fraction in humans and mice: embracing clinical complexity in mouse models. Eur. Heart J..

[23] Liao S., Tang Y., Yue X., Gao R., Yao W., Zhou Y. (2021). Beta-hydroxybutyrate mitigated heart failure with preserved ejection fraction by increasing Treg cells via Nox2/GSK-3beta. J. Inflamm. Res..

[24] Deng Y., Xie M., Li Q., Xu X., Ou W., Zhang Y. (2021). Targeting mitochondria-inflammation circuit by beta-hydroxybutyrate mitigates HFpEF. Circ. Res..

[25] Borlaug B.A., Sharma K., Shah S.J., Ho J.E. (2023). Heart failure with preserved ejection fraction: JACC scientific statement. J. Am. Coll. Cardiol..

